# LyoPRONTO: an Open-Source Lyophilization Process Optimization Tool

**DOI:** 10.1208/s12249-019-1532-7

**Published:** 2019-10-31

**Authors:** Gayathri Shivkumar, Petr S. Kazarin, Andrew D. Strongrich, Alina A. Alexeenko

**Affiliations:** School of Aeronautics and Astronautics, Purdue University, 701 W. Stadium Ave., West Lafayette, IN 47907 USA

**Keywords:** Freeze-drying, Lyophilization, Heat and mass transfer, Quality by design (QBD), Process optimization, Freezing model

## Abstract

This work presents a new user-friendly lyophilization simulation and process optimization tool, freely available under the name LyoPRONTO. This tool comprises freezing and primary drying calculators, a design-space generator, and a primary drying optimizer. The freezing calculator performs 0D lumped capacitance modeling to predict the product temperature variation with time which shows reasonably good agreement with experimental measurements. The primary drying calculator performs 1D heat and mass transfer analysis in a vial and predicts the drying time with an average deviation of 3% from experiments. The calculator is also extended to generate a design space over a range of chamber pressures and shelf temperatures to predict the most optimal setpoints for operation. This optimal setpoint varies with time due to the continuously varying product resistance and is taken into account by the optimizer which provides varying chamber pressure and shelf temperature profiles as a function of time to minimize the primary drying time and thereby, the operational cost. The optimization results in 62% faster primary drying for 5% mannitol and 50% faster primary drying for 5% sucrose solutions when compared with typical cycle conditions. This optimization paves the way for the design of the next generation of lyophilizers which when coupled with accurate sensor networks and control systems can result in self-driving freeze dryers.

**INTRODUCTION**

Lyophilization refers to the energy and time intensive process of solvent (typically water) removal used to improve the long-term storage stability of perishable materials (1,2). A typical pharmaceutical lyophilization cycle is composed of three stages namely, freezing, primary drying, and secondary drying. An optimal lyophilization cycle is one that achieves the highest drug quality for the least cost (3). Of the three stages, primary drying is usually the longest part of the cycle (4) and its optimization shortens the cycle time resulting in a higher throughput for a given lyophilizer and thereby, lower manufacturing cost (3). On the other hand, a non-optimal cycle not only takes longer and costs more than essential but may also compromise drug stability (3).

Developing a better understanding of the freezing process is crucial to lyophilization understanding and optimization because the sublimation rate of ice during primary drying is strongly influenced by the ice crystal morphology. A typical profile of the product temperature during freezing is shown in Fig. 1. The product in the vial is cooled till the nucleation temperature (*T*_n_) is reached and nucleation begins. Due to the latent heat generated by nucleation, the product temperature increases to the freezing temperature (*T*_f_). Beyond this point, the ice crystallization occurs while heat is released till the entire sample is frozen. The temperature decreases continuously during the crystallization process and during the final step of solid cooling.

**Fig. 1:**
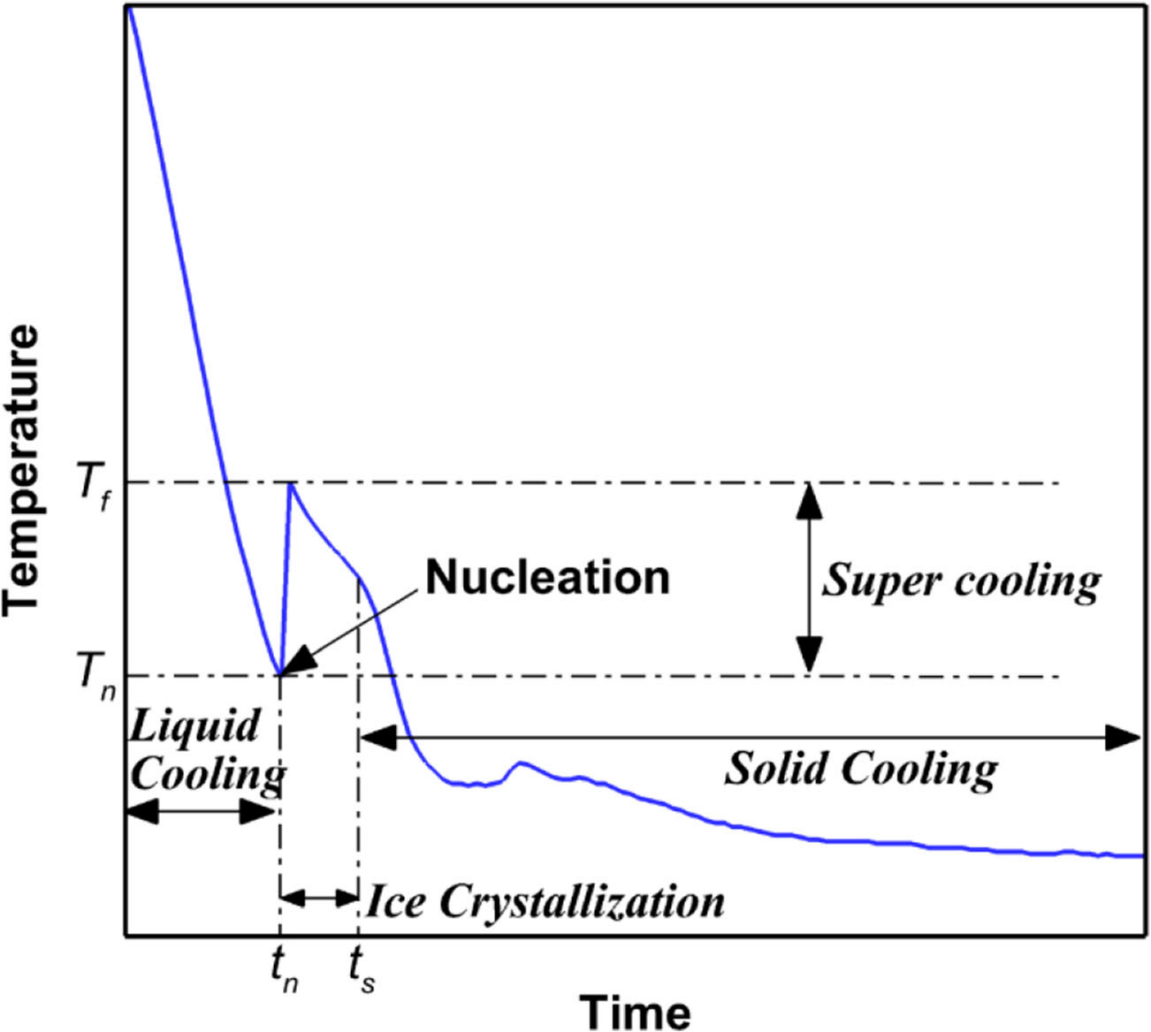
Typical product temperature profile in a lyophilization vial during freezing

Nakagawa *et al.* (5) and Hottot *et al.* (6) developed a two-dimensional finite-element model based on the models proposed by Qin *et al.* (7) and Lunardini (8) for freezing analysis in lyophilization vials. In the present work, we propose a simplified mathematical model of the freezing step using the lumped capacitance method of heat transfer analysis (9). We compare the simulated results with experimental measurements. The simplified freezing calculator is one of the modes of operation of the Lyophilization Process Optimization Tool (LyoPRONTO) (10), written using Python.

The heat and mass transfer through a freeze-drying vial during primary drying have been successfully modeled in the past, and these models show reasonable agreement with experimental measurements (11). The interaction of the primary drying input variables such as process parameters and material attributes which affect the quality of the product can be represented on a design space (12–15), which is a crucial part of the Quality-by-Design (QbD) paradigm (4). The equipment capability limit forms one of the bounding elements of the design space, and the limitation occurs due to sonic flow in the duct (16) or limited condenser refrigeration capacity. The other is formed by the critical product temperature beyond which the product quality is no longer acceptable (12). Figure 2 shows a typical design space with the equipment and product limits, as well as shelf temperature isotherms, on a plot of sublimation flux *versus* the chamber pressure. The yellow region represents the safe design space where it is desirable to operate.

**Fig. 2:**
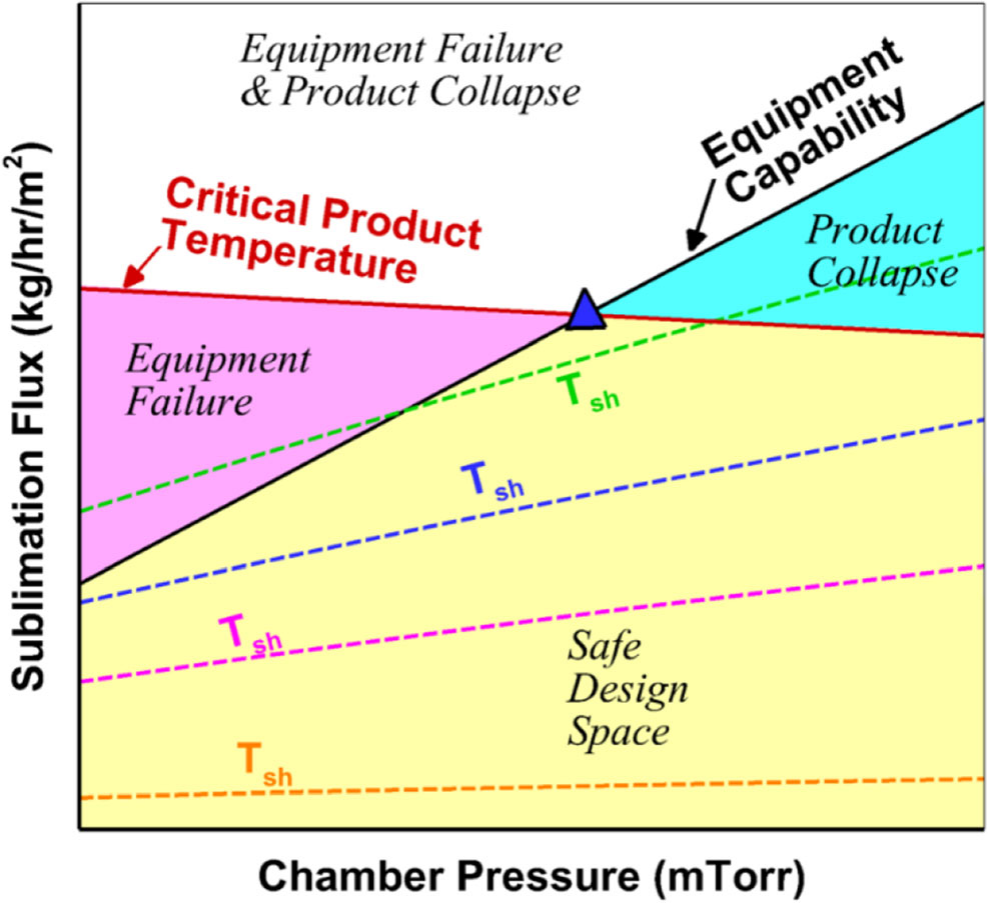
Typical primary drying design space

The current practice in lyophilization is to control the chamber pressure and shelf temperature at constant setpoints based on rules of thumb using open-loop control. The setpoint values are chosen conservatively which leads to an energy efficiency of less than 5% for the cycle. Typically, the chamber pressure is maintained at a setpoint between 50 and 200 mTorr (3). The shelf temperature is maintained at a constant value that ensures that the product temperature does not exceed the target temperature by a predetermined tolerance of 1–2°C at any point during the primary drying process. The target product temperature is taken to be 2–5°C below the critical value beyond which the product appearance is unacceptable (3). The entire freeze-drying process could take days or even weeks to finish when these input conditions are not optimized (2,17).

Although operating at any condition within the yellow region in Fig. 2 is acceptable, the most optimal operating point is at the intersection of the equipment capability curve and the product temperature isotherm corresponding to the maximum allowable product temperature on the design space (12). This point, indicated by the blue triangle, provides the constant setpoints of chamber pressure and shelf temperature for the primary drying for maximum cycle efficiency. However, at every instant during the drying, this point changes due to the variation of product resistance with cake length. The design space is constructed using the largest value of product resistance which occurs towards the end of the cycle when the cake length is maximum. This ensures that the product temperature limit is not exceeded at any point in the cycle. However, varying the chamber pressure and shelf temperature in real time during the drying, instead of maintaining them at constant setpoints, allows for the most optimal operation throughout the drying process and not just at the end, leading to higher energy efficiency.

In this work, we present the capabilities of LyoPRONTO which can be used as a simple lyophilization calculator for the freezing and primary drying steps, as a primary drying design-space generator, and as a process optimizer. The 0D freezing model in the “freezing calculator” mode predicts the time variation of the product temperature during the freezing and the total freezing time. The 1D quasi-steady heat and mass transfer analysis through a vial in the “primary drying calculator” mode predicts the time variation of product temperature and cake length, as well as the total primary drying time when the chamber pressure and shelf temperature setpoints are specified along with the heat transfer and product parameters. In addition, the heat transfer parameters can be determined based on the experimental drying time, and product resistance can be calculated based on the experimental product temperature profile. Moreover, the input chamber pressure and shelf temperature setpoints specified need not stay constant throughout during drying but can be specified as a function of time, and the shelf temperature ramping from freezing or annealing to primary drying is accounted for. The “design-space generator” mode extends this to a range of specified chamber pressure and shelf temperature setpoints. The “optimizer” mode determines the most optimal chamber pressure and/or shelf temperature in real time within their bounding values for given product, load, and equipment characteristics. This method, when coupled with accurate measurements of instantaneous sublimation rates using a network of pressure and/or temperature sensors, can achieve closed-loop control of the process resulting in self-driving freeze dryers.

The remainder of this paper is organized as follows. The next section presents the modeling methodology used in LyoPRONTO. The following section describes the experimental setup used for comparing typical freeze-drying cycles with modeling results. “BENCHMARKING OF THE MODEL” presents the studies used to compare LyoPRONTO results with published modeling and experimental results, as well as our own experimental measurements. Finally, the “PROCESS OPTIMIZATION” outlines the need for variable input parameter lyophilization cycles, and the reduction in primary drying time for 5% mannitol and 5% sucrose formulations when optimized variable chamber pressures and shelf temperatures are used instead of typical cycle setpoints.

## NUMERICAL MODEL

### Freezing Calculator

LyoPRONTO performs a 0D calculation of the transient heat conduction during the freezing process. The model assumptions are (i) lumped capacitance heat transfer, (ii) constant product temperature during ice crystallization, and (iii) heat is transferred to the product only from the shelf. Lumped capacitance method refers to the assumption of low resistance to conduction within the product when compared with the heat transfer between the product and its surroundings and thereby, of a spatially uniform temperature within the product. The temperature gradient within the product is small when the Biot number, *Bi* ≪ 1, and large for *Bi* ≫ 1 (9). Biot number is defined as *Bi* = *hL*_c_/*k* where, *h* is the heat transfer coefficient, *L*_c_ is the characteristic length taken as the maximum of the fill height of the product or diameter of the vial, and *k* is the thermal conductivity of the product. In general, the error associated with the lumped capacitance method is very small for *Bi* < 0.1 (9). During freezing of the product in a vial, the average *Bi* during cooling (based on water) is ~ 0.6, and during crystallization and solid cooling is ~ 0.1. Since these values are slightly greater than the cut-off value, the lumped capacitance method leads to a higher error, but the agreement is taken to be satisfactory owing to the stochastic nature of the nucleation process which leads to a significant error even when the time-intensive finite-element method is used. This can be seen in the work of Nakagawa *et al.* (5), where a reduction in the specified nucleation temperature from − 2.5 to − 4.5°C leads to a 20% reduction in the ice crystallization time, and a further reduction in the specified nucleation temperature from − 4.5 to − 10°C leads to a 34% reduction in the ice crystallization time.

The 0D transient equation for heat transfer used is given by:

1 $$ \rho {C}_{\mathrm{p}}V\frac{\partial {T}_{\mathrm{p}\mathrm{r}}}{\partial t}=-h{A}_v\left({T}_{\mathrm{p}\mathrm{r}}-{T}_{\mathrm{sh}}\right) $$

where, *ρ* is the product density in the liquid form, *C*_p_ is its specific heat capacity, *V* is the product volume, *T*_pr_ is the product temperature, *t* is the time, *h* is the heat transfer coefficient between the shelf and the product, *A*_v_ is the vial area, and *T*_sh_ is the shelf temperature. The negative sign indicated that the product temperature decreases with time. In our model we estimate the heat transfer coefficient based on the experimental cooling rate of the product using Eq. 1 during the initial temperature drop till the nucleation point is reached. The nucleation and freezing temperatures are also obtained from the experimental thermocouple measurements owing to the stochastic nature of the nucleation process when it is not controlled. The heat released during the temperature jump from *T*_n_ to *T*_f_ is used to determine the crystallization time Δ*t*, as:

2 $$ \rho \mathrm{V}\left({H}_{\mathrm{f}}-{C}_{\mathrm{p}}\left({T}_{\mathrm{f}}-{T}_{\mathrm{n}}\right)\right)=h{A}_{\mathrm{v}}\left({T}_{\mathrm{p}\mathrm{r}}-{T}_{\mathrm{sh}}\right)\Delta t $$

where, *H*_f_ is the latent heat of fusion of ice. The temperature is assumed to be constant and equal to *T*_f_ during the crystallization. It is significant to note that this method neglects the heat transfer between the ambient air and the product and only considers heat transfer between the shelf, the vial, and the product. Solid cooling occurs once the crystallization is complete and is modeled using Eq. 1 with material properties corresponding to the solid phase of the product (ice).

A limitation of this model is that experimentally measured nucleation and freezing temperatures must be provided as inputs to the model. Ice nucleation is spontaneous and random, and the nucleation temperature depends on the solution properties, process parameters, environmental factors, container characteristics, and the presence of particulate matter (18). One method of overcoming the large variability in these stochastic parameters is to use a range of typical temperatures at which nucleation and freezing occur based on the literature and previously obtained experimental data. The model provides a range of expected output properties and the process parameters can be modified in order to ensure that the outputs lie in the desired range of values. Controlled nucleation technology (18,19) can also be used to eliminate the uncertainty in the nucleation process and there are multiple methods of achieving this. Seeding the product with inorganic compounds or bacteria, pre-treating the vials to increase the surface roughness, ultrasound nucleation, and vacuum-induced freezing are known methods of controlling nucleation. The ice-fog technique is another popular approach to control nucleation by introducing cold nitrogen gas at pressures close to atmospheric conditions into the chamber to freeze the moisture content. The ice crystals thus formed induce nucleation at the surface of the solution. Using controlled nucleation, it is possible to accurately predict the nucleation temperature and mitigate the requirement of experimental calibration for the model.

### Primary Drying Calculator

LyoPRONTO performs heat and mass transfer modeling to determine primary drying time as well as the sublimation flux and maximum product temperature as functions of time during primary drying. The model assumptions are (i) 1D quasi steady state heat and mass transfer, (ii) center vial is representative of the entire batch, and (iii) convective heat transfer can be neglected. The lyophilization calculator and design-space generator are based on previously published 1D models (4,20–23). We ignore the non-uniformity in heat transfer due to edge effect and consider the center vial to be representative of the entire batch. The heat transfer can thus be approximated as one-dimensional due to symmetry about the central axis of the vial.

**Fig. 3:**
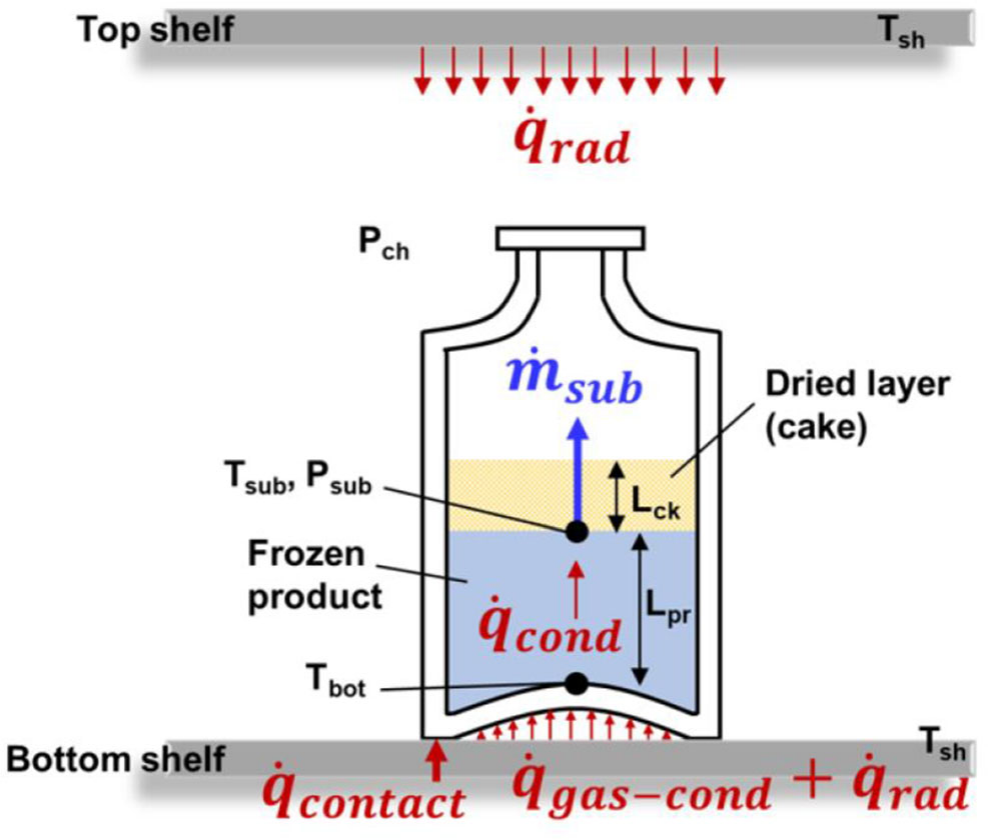
Schematic of the heat and mass transfer in a center vial during primary drying

The heat and mass transfer balances in a vial are shown in Fig. 3. The sources of heat for the product during primary drying are the conductive heat transfer due to contact between the vial bottom and the shelf top surface, the conduction through the gas between the vial bottom and the shelf, and radiative heat transfer from the top and bottom shelves. These are combined together in single vial heat transfer coefficient term (*K*_v_), which is a function of the chamber pressure (*P*_ch_), as given by (20,24,25):

3 $$ {K}_{\mathrm{v}}={K}_{\mathrm{c}}+\frac{K_{\mathrm{p}}{P}_{\mathrm{c}\mathrm{h}}}{1+{K}_{\mathrm{D}}{P}_{ch}} $$

where, *K*_c_, *K*_p_ , and *K*_*D*_ are fitting parameters that depend on the vial and the lyophilizer.

This heat is transferred from the bottom of the product in the vial to the sublimation interface by conduction through the frozen product. The sublimation begins at the product surface exposed to the chamber and proceeds downwards. As the sublimation proceeds, the dried layer length, also called cake length, increases and frozen product length reduces. The total heat flow balance is given by:

4 $$ \dot{Q}=\left({T}_{\mathrm{sh}}-{T}_{\mathrm{bot}}\right){K}_{\mathrm{v}}{A}_{\mathrm{v}}=\frac{\left({T}_{\mathrm{bot}}-{T}_{\mathrm{sub}}\right){A}_{\mathrm{p}}{k}_{\mathrm{ice}}}{L_{pr}}=\frac{\left({P}_{\mathrm{sub}}-{P}_{\mathrm{ch}}\right)\ {A}_{\mathrm{p}}\ \varDelta {H}_s\ }{R_{\mathrm{p}}} $$

where, *T*_sh_ is the shelf temperature, *T*_bot_ is the product bottom temperature, *T*_sub_ and *P*_sub_ are the temperature and pressure at the sublimation interface, *A*_v_ and *A*_p_ are the areas of the vial and the product, *k*_ice_ is the conductivity of the frozen product, and Δ*H*_s_ is the heat of sublimation. *P*_sub_ corresponds to the vapor pressure at *T*_sub_. The product resistance (*R*_p_), is a function of the cake length (*L*_*ck*_, and can be expressed as (26, 27,28):

5 $$ {R}_{\mathrm{p}}={R}_0+\frac{A_1{L}_{\mathrm{ck}}}{1+{A}_2{L}_{\mathrm{ck}}} $$

where, *R*_0_, *A*_1_ , and *A*_2_ are fitting parameters. The heat balance is solved iteratively for small increments in time to determine the sublimation flux and product temperature as a function of the primary drying time at any given chamber pressure and shelf temperature. The iterations continue till the entire product is dried, *i.e.*, the cake length is equal to the initial length of the frozen product. The drying time is the time when the cake length reaches the initial product length (*L*_pr, 0_), which is determined as (11):

6 $$ {L}_{\mathrm{p}\mathrm{r},0}=\frac{V_{\mathrm{fill}}}{A_{\mathrm{p}}{\rho}_{\mathrm{ice}}}\left({\rho}_{\mathrm{solution}}-\frac{c_{\mathrm{solid}}\left({\rho}_{\mathrm{solution}}-{\rho}_{\mathrm{ice}}\right)}{\rho_{\mathrm{solute}}}\right) $$

where, *V*_fill_ is the fill volume, *c*_solid_ is the solute concentration in mass per unit volume units, and *ρ*_ice_, *ρ*_solution_ , and *ρ*_solute_ are the densities of ice, solution, and solute, respectively.

LyoPRONTO also has modes where *K*_v_ and *R*_p_ can be determined if they are unknown. Instead of additional time-consuming experiments to determine the heat transfer characteristics, *K*_v_ is determined by iterating through a range of values to match the calculated drying time with a known experimental drying time for the same input conditions. Moreover, if this can be determined at three or more chamber pressures, a curve fit through the points provides the three coefficients, *K*_c_, *K*_p_ , and *K*_*D*_ for the given vial and lyophilizer combination. If the product temperature profile is known from experiments, this is used to calculate *R*_p_ as a function of cake length, which in turn is curve fit to determine the coefficients, *R*_0_, *A*_1_ , and *A*_2_.

### Design-Space Generator

The same heat and mass transfer calculations are performed by the design-space generator tool to generate the shelf temperature isotherms by performing the calculations at a range of shelf temperature and chamber pressure setpoints. The equipment capability limit is a straight line whose parameters need to be inputted in the form:

7 $$ {\dot{m}}_{\mathrm{eq}\ \mathrm{cap}}=a+b{P}_{\mathrm{ch}} $$

where, *a* and *b* can be determined using experimental choked flow tests (29), minimum controllable pressure tests (30), or using computational fluid dynamics (CFD) modeling of choked flow through a lyophilizer (23,31,32). The critical product temperature isotherm is determined by fixing the vial bottom temperature at the maximum allowable value (*T*_pr, max_), which is typically taken to be 2 to 3°C below the critical temperature. In this case, the chamber pressure is fixed, but the shelf temperature is not. The sublimation front temperature, and thereby the sublimation rate, at every time step are determined based on the fixed *T*_bot_. It is worth noting that LyoPRONTO accounts for the shelf temperature ramping from the freezing to the primary drying stages based on the ramping rate and freezing shelf temperature provided.

### Optimizer

**Fig. 4:**
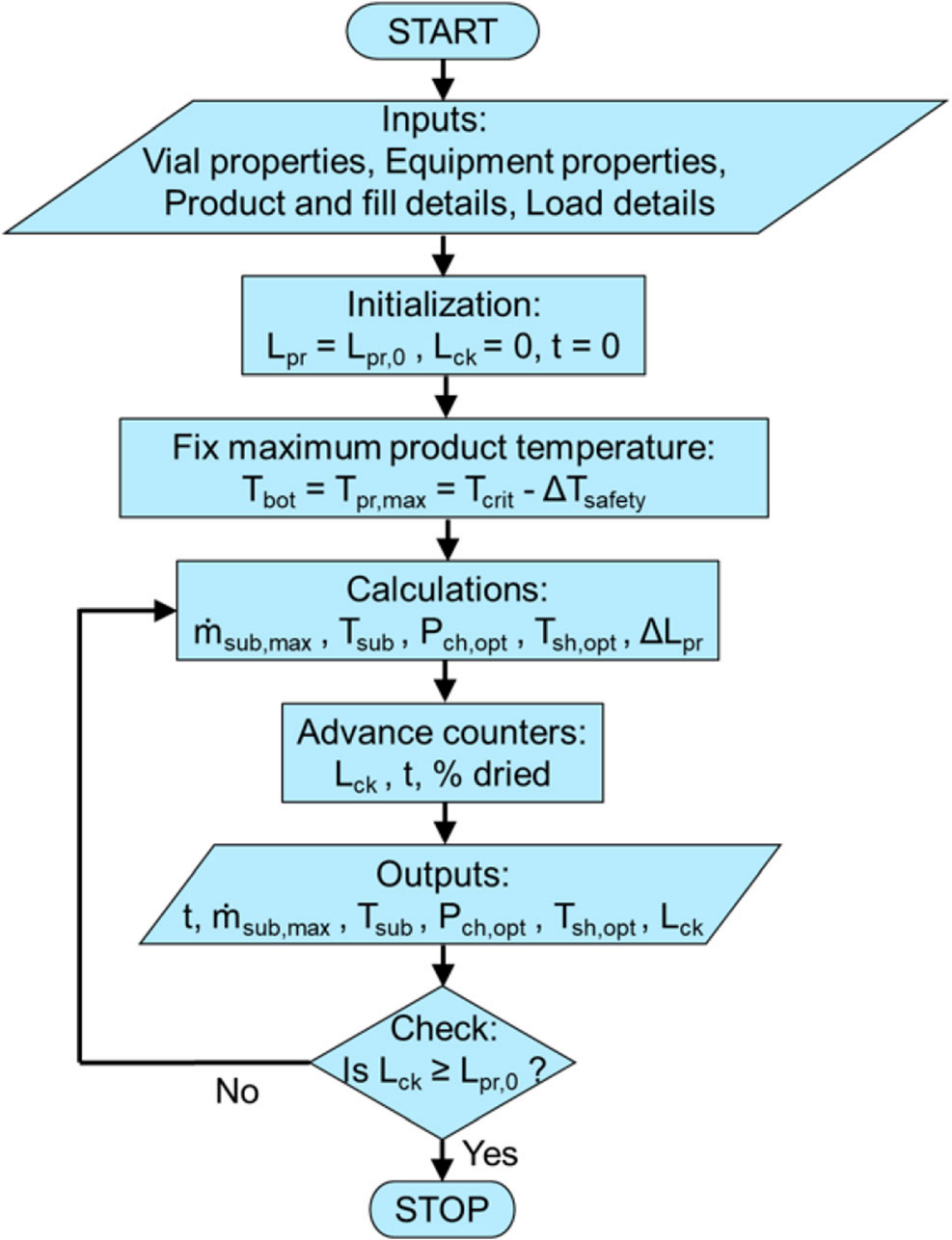
LyoPRONTO algorithm for the “Optimizer” mode

The key new feature of LyoPRONTO is the optimizer tool which determines the optimal chamber pressure and shelf temperature at each time step such that the total drying time is minimized. For given vial, lyophilizer, product, and load parameters, the maximum possible sublimation flux is determined based on Eq. 4 within the constraints ${\dot{m}}_{\mathrm{tot}}\le {\dot{m}}_{\mathrm{eq}\ \mathrm{cap}}$ and *T*_bot_ ≤ *T*_pr, max_ where, ${\dot{m}}_{\mathrm{tot}}$ is the total sublimation rate of all the loaded vials. The optimal parameters *P*_ch, opt_ and *T*_sh, opt_ are the ones that result in this maximum possible sublimation flux which are determined iteratively at each time step till the drying is complete. Figure 4 summarizes these steps in a flowchart. In addition to the equipment and product constraints, maximum and minimum constraints can be imposed on the chamber pressure and shelf temperature based on practical limitations such as shelf fluid properties, vial glass properties, freeze-drying chamber leakage, and vacuum pump capacity. Optimization can also be performed for just one of the two parameters, *P*_ch_ and *T*_sh_, while keeping the other fixed at specified setpoints. The effect of varying and optimizing both or just one of the two cycle parameters is explored in the optimization section.

## EXPERIMENTAL METHODOLOGY

We perform experiments at constant chamber pressure and shelf temperature setpoints for comparison with the modeling results in order to evaluate the performance of LyoPRONTO. We use the laboratory-scale freeze-dryer REVO by Millrock Technology, Kingston, NY for our experiments. One of the four loadable shelves of the lyophilizer is loaded fully with 398 Schott 6R vials filled with 2 mL of 5% mannitol solution each. For the freezing step, we ramp the shelf temperature from room temperature to − 20°C at a rate of 1°C/min and hold for 2 h. Next, we ramp the shelf temperature to − 5°C and hold for 1 h. The primary drying is carried out at a shelf temperature of − 5°C with the chamber pressure in the 100 to 1500 mTorr range. The experiment at 300 mTorr is repeated for the same conditions using pure water as the product in order to evaluate the performance of the lumped capacitance freezing model.

The convergence of the Pirani gauge measurement with the capacitance manometer (CM) reading marks the end of primary drying for the center vials (33). Separate experiments are not performed to determine the heat transfer and product resistance parameters. LyoPRONTO is used to determine the heat transfer characteristics of the 6R vials in REVO which are presented in the following section. Since, the product resistance of 5% mannitol with uncontrolled nucleation is well documented in the literature (11) and we use these values given in Table I.

**Table I:** Product Resistance Parameters According to Eq. 5 for Products Modeled Here

Product	*R*_0_		*A*_1_		*A*_2_	
	× 10^4^ m/s	cm^2^ h Torr/g	× 10^7^ s^−1^	cm h Torr/g	× 10^2^ m^−1^	cm^−1^
5% mannitol (11)	6.72	1.4	7.68	16	0	0
5% povidone (11)	5.42	1.13	2.40	5	0	0
0.5% lysozyme at *T*_sh_ = − 25 ° C (34)	2.23	0.4638	9.89	20.6	4.394	4.394
0.5% lysozyme at *T*_sh_ = 25 ° C (34)	5.98	1.2467	0	0	0	0
2% lysozyme at *T*_sh_ = − 25 ° C (34)	3.98	0.829	7.76	16.16	2.386	2.386
2% lysozyme at *T*_sh_ = 25 ° C (34)	11.77	2.4518	0	0	0	0
0.5% BSA at *T*_sh_ = − 25 ° C (34)	2.60	0.5429	9.94	20.7	4.951	4.951
0.5% BSA at *T*_sh_ = 25 ° C (34)	6.53	1.3601	0	0	0	0
2% BSA at *T*_sh_ = − 25 ° C (34)	5.43	1.131	9.79	20.4	0.1019	0.1019
2% BSA at *T*_sh_ = 25 ° C (34)	14.61	3.043	0	0	0	0
0.5% IgG at *T*_sh_ = − 25 ° C (34)	4.42	0.9204	5.23	10.9	1.349	1.349
0.5% IgG at *T*_sh_ = 25 ° C (34)	7.00	1.4582	0	0	0	0
2% IgG at *T*_sh_ = − 25 ° C (34)	4.15	0.8646	10.79	22.49	1.716	1.716
2% IgG at *T*_sh_ = 25 ° C (34)	15.36	3.2	0	0	0	0
5% sucrose (22)	1.00	0.208	7.34	15.29	1.6	1.6

## BENCHMARKING OF THE MODEL

### Comparison with Experimental Measurements

#### Freezing

**Fig. 5:**
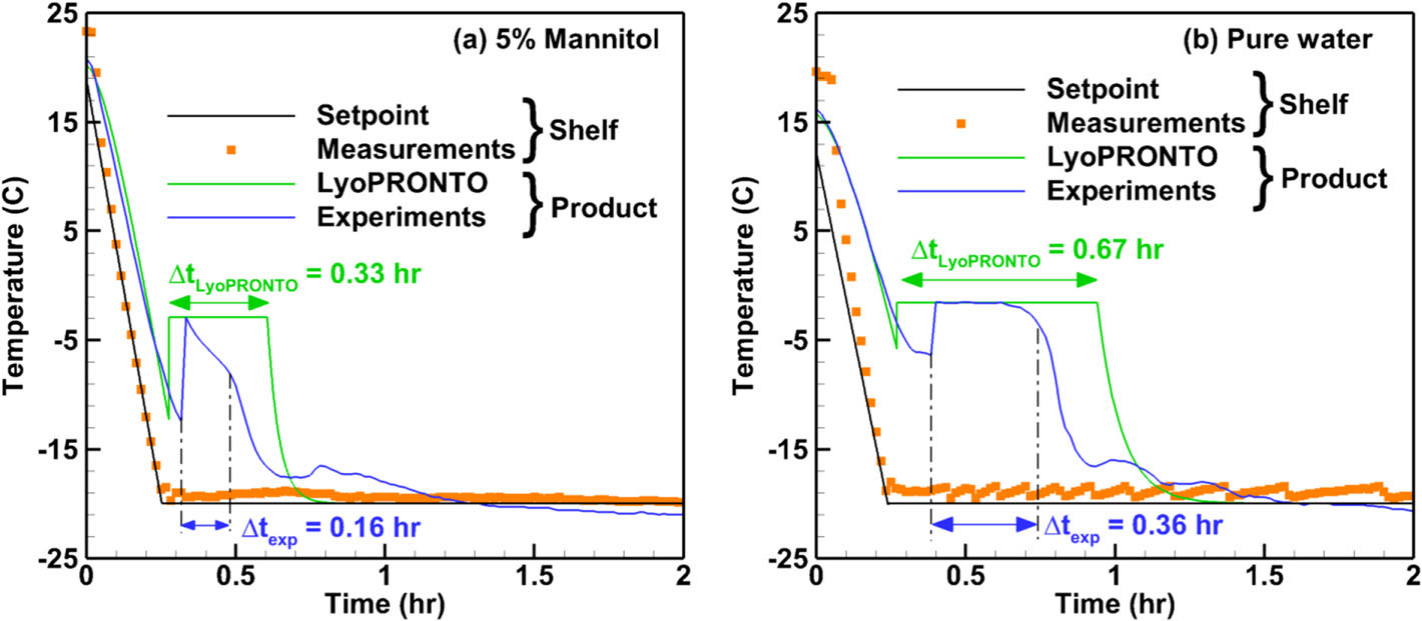
Comparison of LyoPRONTO and experimental temperature profiles during the freezing of 2 mL of **a** 5% mannitol and **b** pure water in 6R vials in REVO

Figure 5 compares the product temperature profile predicted by the lumped capacitance model with experimental thermocouple measurements at the vial bottom for the freezing of 5% mannitol solution and pure water. Figure 5 also shows the shelf temperature setpoint and the shelf surface temperature measured by the heat flux sensor built into the lyophilizer shelf. The heat transfer coefficient is determined based on a nonlinear least squares curve fit for the thermocouple measurements and the best fit provides a 20% lower value of nucleation onset time on average when compared with the experimental value.

The crystallization and solid cooling steps can be clearly distinguished in the product temperature profiles. The simulated crystallization time is about twice the experimental value for both cases due to the assumption of lumped capacitance. The product temperature profiles during solid cooling obtained using LyoPRONTO and thermocouples display similar trends and converge to the shelf temperature by the end of the solid cooling process. The fluctuation in the measured product temperature profile near the shelf temperature setpoint value towards the end of solid cooling is expected to be due to the variation in the shelf surface temperature itself as shown by the measured shelf temperature profile. While setting up a freezing process during a freeze-drying cycle, it is required to specify the time for which the shelf temperature must be maintained at the value corresponding to the freezing step. Beyond this time, the setpoints are changed to the annealing or primary drying step values. If a range of expected *T*_f_ and *T*_n_ values are known based on previous experiments, the freezing model can predict a range of times required for the completion of the freezing process. Since the model overpredicts the crystallization time when compared with the experiments, it is safe to use these values to predict the total time required for the freezing step. Since the uncontrolled nucleation process is stochastic, the agreement between the model and experiments can be taken to be satisfactory when designing a freezing cycle where a factor of safety can be incorporated. This model has the added advantage of being user friendly, computationally less expensive than a multi-dimensional finite element model, and fast.

#### Primary Drying

**Fig. 6:**
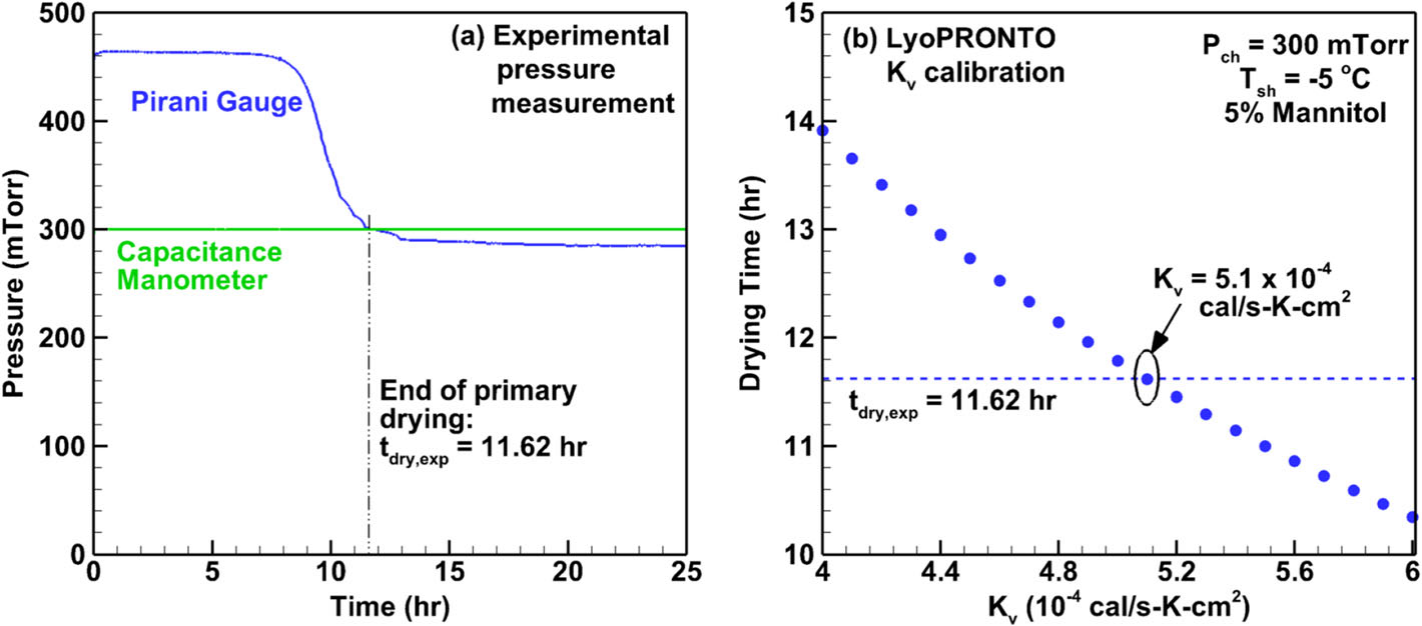
**a** Primary drying end time determination based on experimental pressure measurements and **b** *K*_v_ calibration at *P*_ch_ = 150 mTorr, *T*_sh_ = − 5°C for 2 mL of 5% mannitol in 6R vials in REVO

We perform experimental lyophilization runs at chamber pressures of 100, 300, and 1500 mTorr and a shelf temperature of − 5°C as described in the “EXPERIMENTAL METHODOLOGY” section, and the primary drying time is found based on the convergence of the Pirani and manometer readings. An example of this is shown in Fig. 6a for the 300 mTorr case for which the drying time is 11.62 h. The primary drying calculator in LyoPRONTO performs calculations for a range of *K*_v_ values to obtain the best agreement between the experimental and simulated drying times. An example of this corresponding to the 300 mTorr case is shown in Fig. 6b. The best agreement with experimental drying time is obtained for *K*_v_ of 5.1 × 10^−4^ cal/s K cm^2^ which gives a simulated drying time of 11.62 h. Thus, the heat transfer coefficient at a chamber pressure of 300 mTorr for the vial and lyophilizer combination is 5.1 × 10^−4^ cal/s K cm^2^ (21.34 W/m^2^ K). Similarly, the values of *K*_v_ that best match the experimental drying time based on Pirani gauge and manometer convergence are found at 100 and 1500 mTorr. Table II shows the results for the best *K*_v_ values at all three chamber pressures and compares the resulting drying time with the experimental value. This provides us with three values of *P*_ch_ and three values of *K*_v_ and the three parameters, *K*_C_, *K*_P_ , and *K*_*D*_ in Eq. 3 are obtained by regression analysis curve fitting. These coefficients are provided in Table III.

**Table II:** Schott 6R Vial Heat Transfer Coefficient in REVO Based on Experimental Drying Time at Different Chamber Pressures

Chamber pressure (mTorr)	Experimental drying time (h)	Simulated drying time (h)	*K*_v_	
			W/m^2^ K	10^−4^ cal/s K cm^2^
100	12.82	12.81	15.06	3.6
300	11.62	11.62	21.34	5.1
1500	15.84	15.84	44.64	10.67

**Table III:** Heat Transfer Parameters According to Eq. 3 for Different Vial/Tray and Lyophilizer Combinations

Vial/tray	Lyophilizer	Heat transfer coefficients
Schott 6R	Millrock REVO	*K*_c_ = 11.51 W/m^2^ K = 2.75 × 10^−4^cal/s K cm^2^ *K*_p_ = 0.28 W/m^2^ K Pa = 8.93 × 10^−4^cal/s K cm^2^ Torr *K*_D_ = 3.45 × 10^−3^Pa^−1^ = 0.46 Torr^−1^
5800 W (11)	Highly modified commercial laboratory-scale lyophilizer	*K*_c_ = 11.04 W/m^2^ K = 2.64 × 10^−4^cal/s K cm^2^ *K*_p_ = 1.03 W/m^2^ K Pa = 33.2 × 10^−4^cal/s K cm^2^ Torr *K*_D_ = 27.3 × 10^−3^Pa^−1^ = 3.64 Torr^−1^
5816 W (11)		*K*_c_ = 8.49 W/m^2^ K = 2.03 × 10^−4^cal/s K cm^2^ *K*_p_ = 1.03 W/m^2^ K Pa = 33.2 × 10^−4^cal/s K cm^2^ Torr *K*_D_ = 29.8 × 10^−3^Pa^−1^ = 3.97 Torr^−1^
5303 (11)		*K*_c_ = 6.36 W/m^2^ K = 1.52 × 10^−4^cal/s K cm^2^ *K*_p_ = 1.03 W/m^2^ K Pa = 33.2 × 10^−4^cal/s K cm^2^ Torr *K*_D_ = 52.3 × 10^−3^Pa^−1^ = 6.97 Torr^−1^
Warped stainless steel tray (11)		*K*_Tc_ = 2.51 W/m^2^ K = 0.6 × 10^−4^cal/s K cm^2^ *K*_Tp_ = 2.04 W/m^2^ K Pa = 65.9 × 10^−4^cal/s K cm^2^ Torr *K*_TD_ = 0.2 Pa^−1^ = 27 Torr^−1^
Wheaton Science 2 mL type-1 tubing vials (34)	SP Scientific LyoStar II	For *P*_ch_ = 57 mTorr and *T*_sh_ = − 25 ° C Using thermocouple measurements: *K*_v_ = 15.9 W/m^2^ K = 3.8 × 10^−4^cal/s K cm^2^ Using Pirani gauge measurements: *K*_v_ = 11.3 W/m^2^ K = 2.7 × 10^−4^cal/s K cm^2^ For *P*_ch_ = 57 mTorr and *T*_sh_ = 25 ° *C* Using thermocouple measurements: *K*_v_ = 8.37 W/m^2^ K = 2.0 × 10^−4^cal/s K cm^2^ Using Pirani gauge measurements: *K*_v_ = 7.95 W/m^2^ K = 1.9 × 10^−4^cal/s K cm^2^

**Fig. 7:**
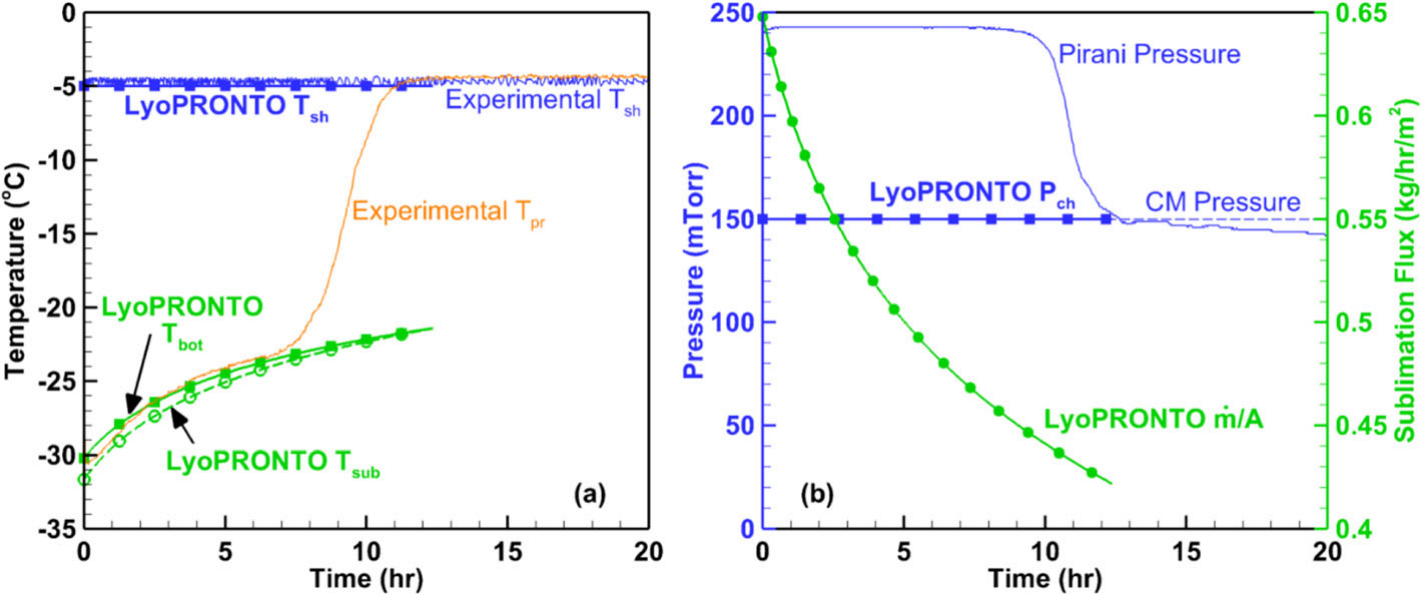
Comparison of LyoPRONTO results with experimental measurements at *P*_ch_ = 150 mTorr, *T*_sh_ = − 5°C for the primary drying of 2 mL of 5% mannitol in 6R vials in REVO

We use the fit heat transfer parameters, *K*_C_, *K*_P_ , and *K*_D_, to determine the *K*_v_ at 150 mTorr. The results modeled using this *K*_v_ are compared with experimental measurements at a chamber pressure of 150 mTorr and shelf temperature of − 5°C in Fig. 7. The experimentally measured parameters are shelf temperature, product temperature at the bottom of the vial, Pirani gauge pressure, and capacitance manometer pressure. The fitted parameters *K*_C_, *K*_P_ , and *K*_D_ are used in LyoPRONTO along with the shelf temperature and chamber pressure setpoints as inputs and the outputs of the simulation are the product temperatures at the vial bottom and sublimation front, and the sublimation flux. The time step used for all the simulations is 3 min. The effect of the time step size is explored in a subsequent section of this paper. The simulated drying time of 12.36 h agrees within 2% of the experimental drying time of 12.62 h. The experimental product temperature at the vial bottom is averaged over three center vials and shows good agreement with the simulated temperature at the same location. The sublimation flux displays the expected trend of decreasing with time due to increasing cake resistance as the drying progresses.

### Comparison with Published Data

Pikal’s seminal paper in 1985 (11) on simulating one-dimensional heat and mass transfer modeling in a vial during primary drying paved the way for the concept of the lyophilization calculator which is widely used in the freeze-drying community today. Pikal studied the effect of shelf temperature and chamber pressure variation on the primary drying time and maximum product temperature for 5% mannitol solution in a variety of vials loaded with and without a tray using the theoretical heat transfer model. The coefficients required to calculate *K*_v_ and *R*_p_ based on Eqs. 1 and 3, respectively, are provided in the paper and summarized in Tables I and III. Table IV provides the geometry parameters for the different vials used here. We use the Pikal’s results to benchmark LyoPRONTO for the same input conditions.

**Table IV:** Geometry Parameters for the Vials used in This Work

Vial	Outer diameter (mm)	Inner diameter (mm)
Schott 6R	22	20
5800W (11)	24.5	22
5816W (11)	29.5	27
5303 (11)	46.8	42.7
Wheaton Science 2 mL type-1 tubing vials (34)	15	13

**Fig. 8:**
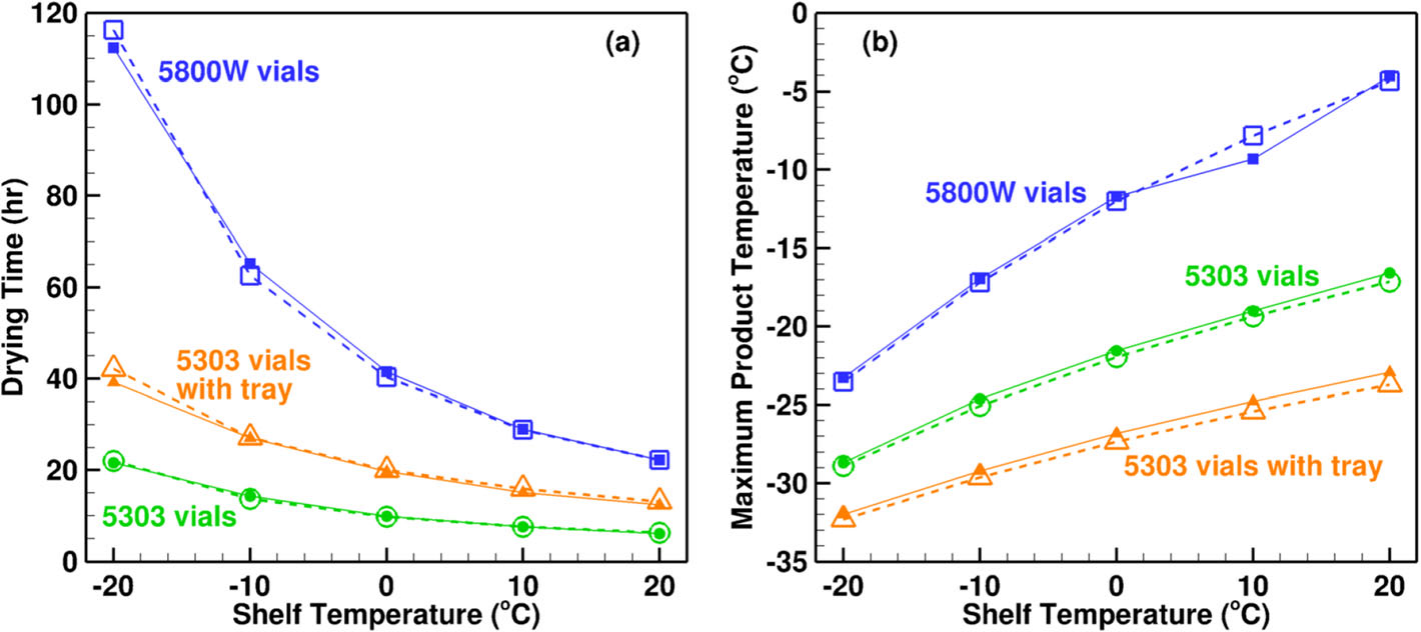
Comparison of LyoPRONTO results (filled symbols, solid lines) with Pikal’s (11) theoretical model predictions (empty symbols, dashed lines) for the primary drying of 8 mL of 5% mannitol at *P*_ch_ = 100 mTorr

**Fig. 9:**
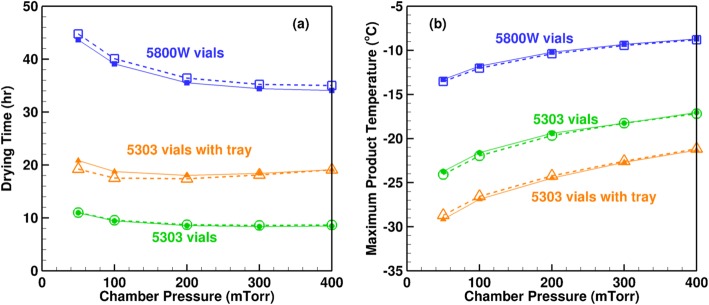
Comparison of LyoPRONTO results (filled symbols, solid lines) with Pikal’s (11) theoretical model predictions (empty symbols, dashed lines) for the primary drying of 8 mL of 5% mannitol at *T*_sh_ = 0°C

Figure 8 compares the variation of drying time and maximum product temperature with the shelf temperature obtained from LyoPRONTO with Pikal’s results. The vials used are 5816 W tubing and 5303 molded vials, and the latter are loaded with and without a warped stainless steel tray at the bottom. Eight milliliters of 5% mannitol is filled in each vial, and the calculations are performed at a chamber pressure of 100 mTorr. From the figure, we see that our results match closely with Pikal’s theoretical model (11) with an average deviation of 1%. The 5303 vials have lower drying times than the 5800-W vials due to their larger area which leads to a lower fill height and thus, lower product resistance even though their *K*_v_ and consequently, the product temperatures, are lower. The inclusion of the tray reduces the heat transferred from the shelf to the vials, and increases the drying time. The cause for the dip in Pikal’s (11) product temperature for the 5800 W vials at 10°C seen in Fig. 8b is unknown and is not seen in the LyoPRONTO results. Figure 9 shows the dependence of the drying time and the maximum product temperature on the chamber pressure for the same product, vial and fill conditions at a shelf temperature of 0°C. The LyoPRONTO results agree well with the published results within an average deviation of 0.3%.

**Fig. 10:**
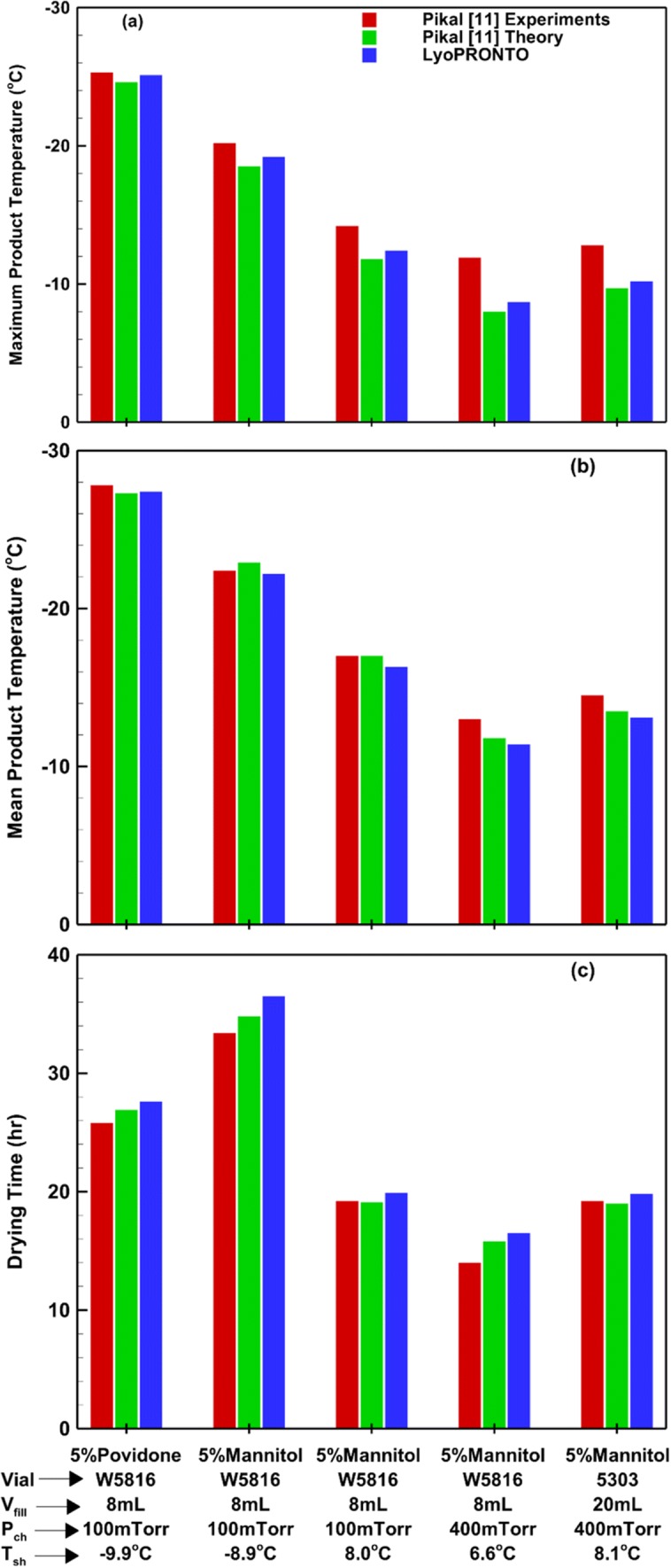
Comparison of LyoPRONTO results with Pikal’s (11) theoretical model predictions and experimental measurements for various primary drying cycles

Pikal’s (11) work also compares the theoretical results with experimental measurements for 5816 W tubing and 5303 molded vials loaded without trays and filled with 5% mannitol or 5% povidone solutions. The fill volume is either 8 or 20 mL. We use LyoPRONTO to simulate the primary drying for these set of inputs and the comparison with the published theoretical and experimental results are shown in Fig. 10. The chamber pressure and shelf temperature for each condition is shown in the figure. The mean product temperature refers to the average *T*_bot_ throughout the cycle. The average deviation between the LyoPRONTO results and the experimental measurements is 4%. The highest deviation for both Pikal’s theoretical and LyoPRONTO results is observed for the maximum product temperature at a chamber pressure of 400 mTorr. For most cases, LyoPRONTO shows better agreement with experimental product temperature measurements on account of a smaller time step, when compared with Pikal’s theoretical model which uses just five divisions of the product length. The time step convergence is studied in more detail in the following subsection.

**Fig. 11:**
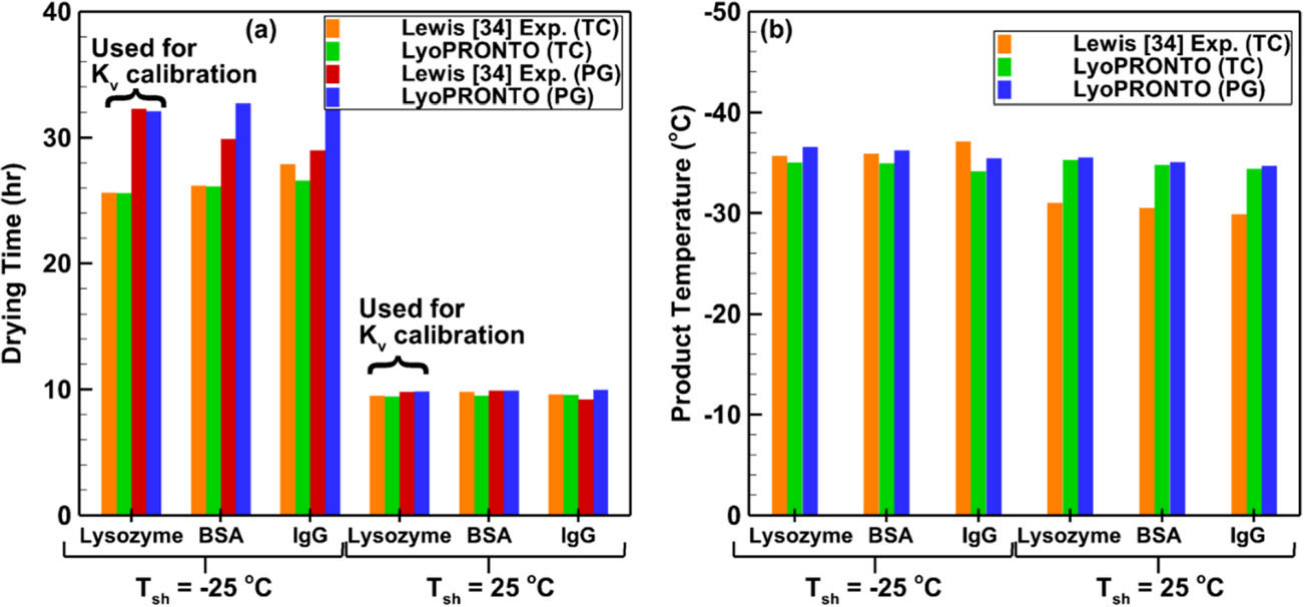
Comparison of LyoPRONTO results with Lewis *et al.’s* (32) experimental measurements for the primary drying of 1 mL of 0.5% protein solution at *P*_ch_ = 57 mTorr

Lewis *et al.* (34) experimentally characterized the primary drying behavior of model protein formulations. They used Wheaton Science 2 mL type-1 tubing vials filled with 1 mL of protein solutions which contained 2.5% sucrose in a 6 mM sodium phosphate buffer of pH 7.4. The primary drying was performed at a chamber pressure setpoint of 57 mTorr and shelf temperature setpoints of − 25°C or 25°C. The product resistance for each of the protein formulations under both typical (*T*_sh_ = − 25°C) and aggressive (*T*_sh_ = 25°C) cycle conditions are provided in the paper and summarized in Table I. The drying time was determined based on both Pirani gauge (PG) convergence with capacitance manometer pressure as well as thermocouple (TC) measurements of product temperature. Since the vial heat transfer parameters were not studied in this work, we extract the *K*_v_ value for the vial at 57 mTorr from the drying time values based on the two aforementioned methods. This is done for the lysozyme-drying time data, and since the vial heat transfer characteristics are independent of the product, we use these values for other protein formulations. Since the radiative heat transfer from the bottom and top shelves to the vials vary significantly at − 25 and 25°C, we obtain two sets of vial heat transfer coefficients based on the shelf temperature and their values are given in Table III. Figure 11 shows the comparison between LyoPRONTO results and experimental measurements of drying time and product temperature for 0.5% protein concentration. The predicted drying times agree very well with experimental measurements, especially at higher shelf temperatures, and the average deviation between the two is found to be 2%. The product temperature results agree within an average deviation of 6% from thermocouple measurements at the bottom of the vial. The *K*_v_ values determined from PG and TC drying times produce product temperature measurements that do not differ significantly from each other, and both consistently overpredict the values at 25°C shelf temperature.

**Fig. 12:**
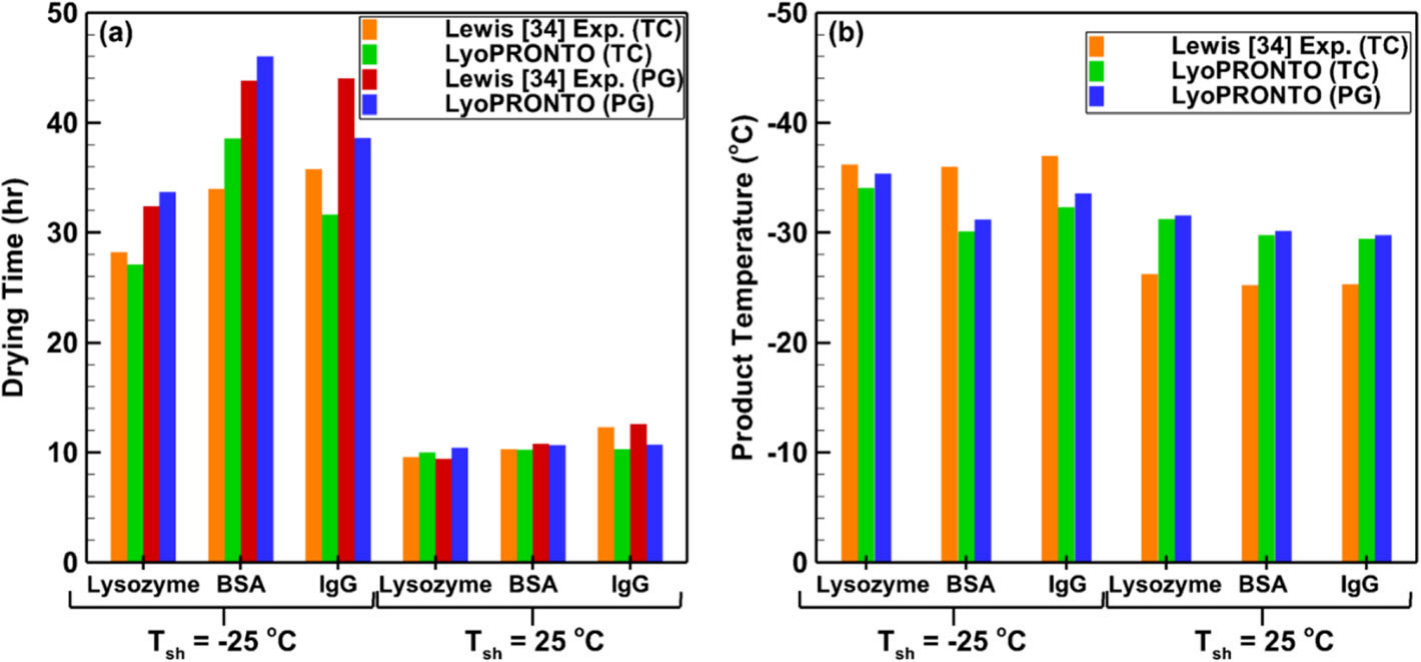
Comparison of LyoPRONTO results with Lewis *et al.’s* (32) experimental measurements for the primary drying of 1 mL of 2% protein solution at *P*_ch_ = 57 mTorr

Figure 12 shows the results for 2% protein concentration for the same set of conditions. Since the heat transfer to the vial is independent of the product formulation, the set of *K*_v_ values determined for 0.5% protein concentration are used for these cases. The predicted and experimental drying times show good agreement once again, with an average deviation of 2%. The modeled product temperatures deviate lesser from the experimental measurements showing an average deviation of 4%. Thus, LyoPRONTO displays the ability to simulate well the primary drying parameters for various products at a wide variety of input conditions.

### Effect of the Iterative Method

The popular iterative method among a majority of the existing lyophilization calculators in the pharmaceutical community is to divide the product length equally into a fixed number of divisions, and perform calculations from a product length of zero to the maximum. In general, the number of divisions used is 5 or 10, and in some rare cases goes up to 100. On the other hand, LyoPRONTO uses the time stepping method to proceed through the primary drying process. The time starts at 0 and proceeds till the entire length of the product is dried in steps which can be specified as an input.

**Fig. 13:**
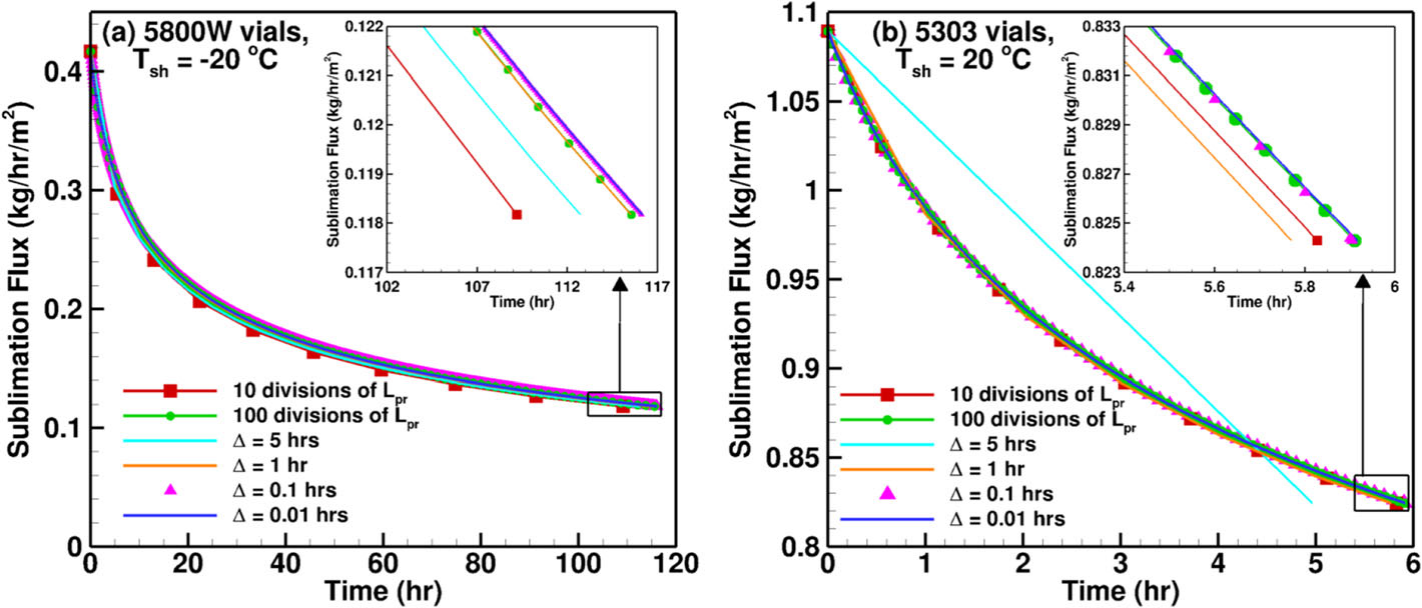
Effect of the iterative method and iterative step refinement on the primary drying modeling of 8 mL of 5% mannitol at *P*_ch_ = 100 mTorr for **a** slow and **b** fast cycles

Figure 13 shows a comparison between the two methods for different number of product length divisions and time step sizes for the primary drying of 8 mL of 5% mannitol at 100 mTorr chamber pressure similar to the conditions shown in Fig. 8. Figure 13a shows a comparison of the results for 5800 W vials at a shelf temperature of − 20°C and Fig. 13b represents drying in 5303 vials at a shelf temperature of 20°C. The former is a slower cycle with lower sublimation rates owing to the lower shelf temperature and smaller product area. When the number of divisions of the product length is increased from 10 to 100, the drying time increases by 7 h. This is because if just 10 divisions are used, a total drying time of 120 h would lead to an assumption of an approximately constant sublimation rate for every 12 h. However, this is not representative of the actual conditions in a lyophilizer where the sublimation rate changes at every instant of time. For the time step variation, we see that a time step of 1 h produces results that are very close to the 100 product length divisions case as expected. Reducing the time step further by an order of magnitude reduces the drying time by 0.5 h (0.4% of drying time) and no significant change is observed when the time step is further reduced to 0.01 h. Thus, the solution is converged for a time step of 1 h.

For the faster cycle shown in Fig. 13b, the drying time is less than 6 h and assuming a time step of 5 h leads to a 16% underestimation of the drying time. However, the solutions are converged for the 1 h time step case as well as the ten product length divisions case. This is because ten divisions produce time steps of approximately 0.6 h. Thus, it is essential to verify whether time step convergence has been achieved for each new cycle condition that is simulated. For the freeze drying of products with low critical temperatures using manufacturing scale lyophilizers, the cycle length may be very large and using 10 or sometimes even 100 divisions of the product length might be insufficient for a converged solution. LyoPRONTO allows for the specification of the time step as an input and the default value used is 0.05 h (3 min). The added advantage of using time stepping instead of product length stepping is the ability to specify the chamber pressure and shelf temperature as functions of time in order to evaluate their effect on the primary drying characteristics.

## PROCESS OPTIMIZATION

### Design-Space Variation During a Cycle

**Fig. 14:**
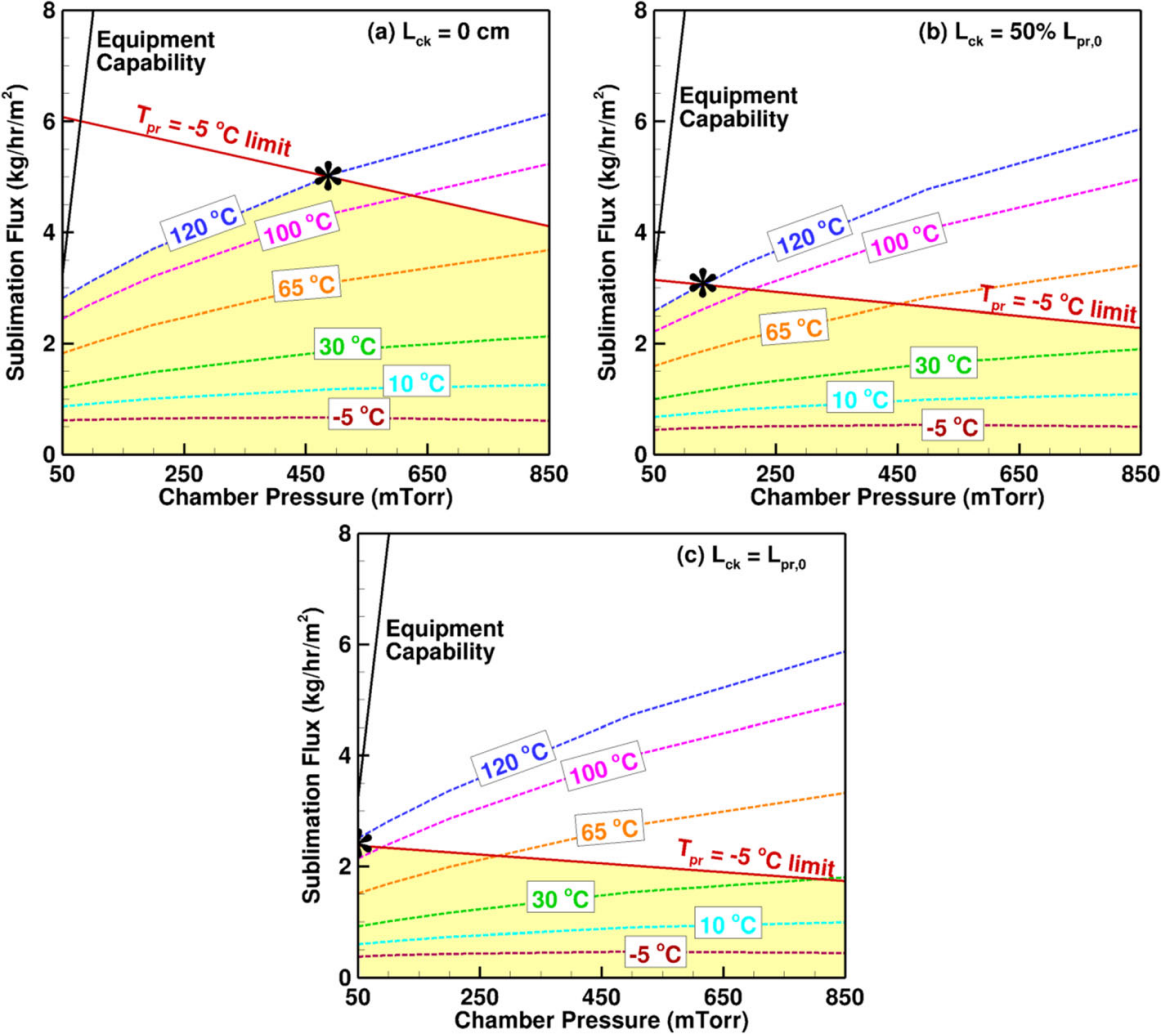
Time variation of the primary drying design space and optimal operating conditions for 2 mL of 5% mannitol in 6R vials on one loaded shelf of REVO

We generate the design space for a range of chamber pressure and shelf temperature setpoints for the lyophilizer, product, and fill and load conditions in Fig. 7. Typically, the design space is generated at the end of primary drying when the product resistance has the highest value in order to ensure that the equipment and product limits are not exceeded at any point during the primary drying. Figure 14 shows the design space at different points during the primary drying for the same vial, fill, equipment, and product parameters as Fig. 7. The major limiting factors are the equipment capability determined using CFD as described by Shivkumar *et al.* (32) and maximum allowable product temperature of − 5°C. We also limit the maximum shelf temperature to 120°C to ensure that the vial and stopper materials remain intact. Since only one of the four shelves is loaded, the total sublimation rate does not reach the high value required to choke the flow in the duct between the chamber and the condenser. Thus, this case is more limited by the product and shelf temperatures, and not by the equipment limit.

When the drying begins, and the cake length is 0, the most optimal chamber pressure at the maximum shelf temperature of 120°C is 480 mTorr. This value falls to 120 mTorr when half the drying is complete, and the cake length is equal to half the initial product length. Towards the end of drying, the inputs are purely limited by the maximum product temperature and the most optimal values are the minimum chamber pressure of 50 mTorr beyond which leakage may occur in the chamber, and a shelf temperature of 110°C. This shows that the optimal primary drying parameters varies significantly and continuously throughout the process and that it is imperative to optimize them in real time in order to improve the process time and energy efficiency.

### Cycle Optimization with Variable Inputs

**Fig. 15:**
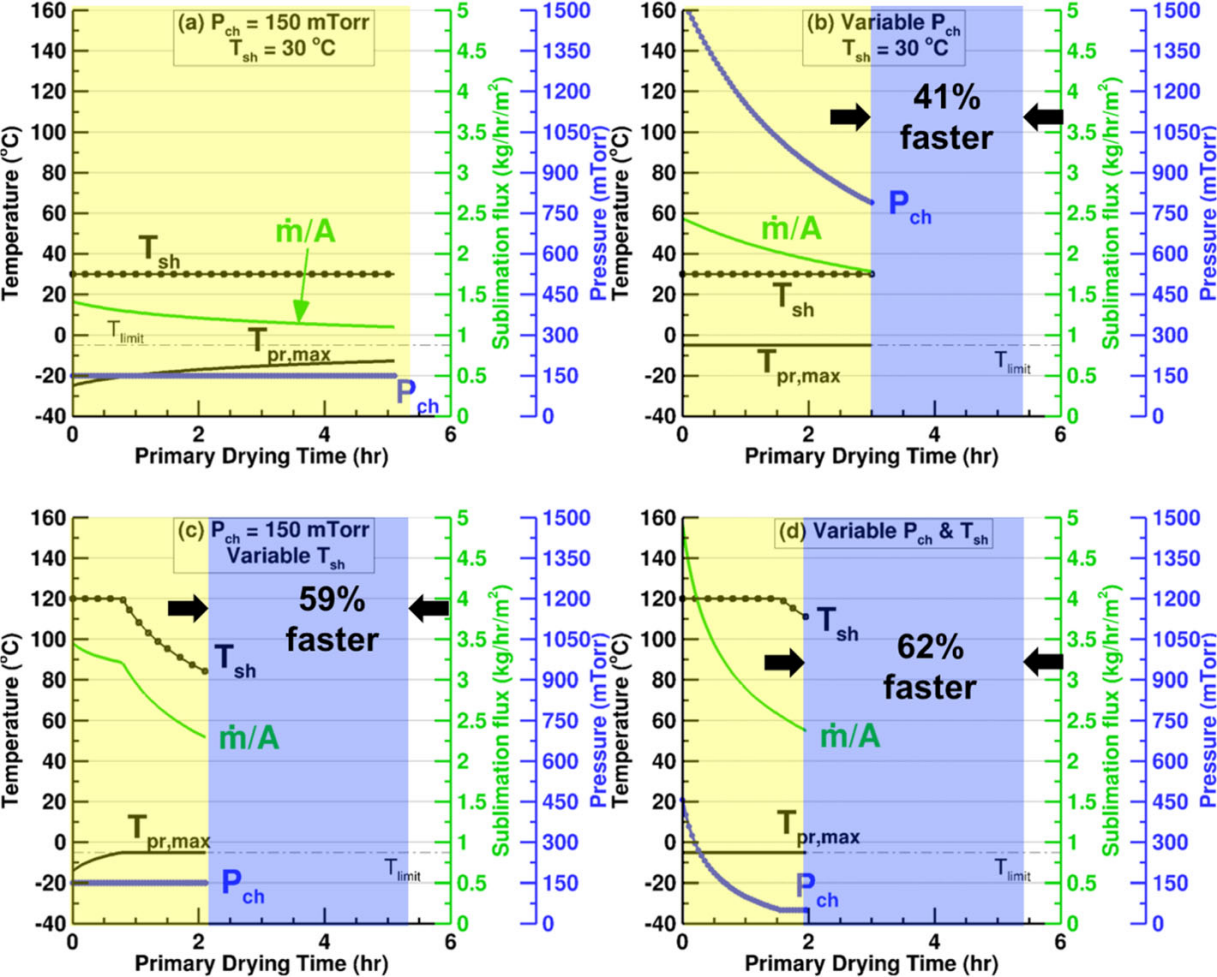
Effect of the *P*_ch_ and/or *T*_sh_ optimization on the primary drying parameters for 2 mL of 5% mannitol in 6R vials on one loaded shelf of REVO

In this section, we compare traditional single setpoint cycles for chamber pressure and shelf temperature to optimized cycles with variable chamber pressure and/or shelf temperature. For the 2 mL of 5% mannitol solution in Schott 6R vials used in the experiments presented earlier, a typical cycle is simulated at the chamber pressure and shelf temperature setpoint values of 150 mTorr and 30°C respectively, as provided by Sane *et al.* (35) for 5% mannitol. Figure 15a shows the results for a partial load cycle with 398 vials on a single shelf of Millrock REVO.

The constraints imposed for the optimized cycle are the maximum product temperature of − 5°C and equipment capability as described in the previous subsection. The maximum shelf temperature and minimum chamber pressure are limited to 120°C and 50 mTorr, respectively. The primary drying time for the typical cycle is 5.11 h. As seen in Fig. 14, these conditions are not the most optimal values and the sublimation flux could be improved significantly by maintaining the cycle parameters at their optimal values. This can also be seen in the maximum product temperature in Fig. 15a which is 12 to 30°C below the limiting value throughout the cycle. Figure 15b shows the results for a variable pressure cycle when the shelf temperature is maintained at 30°C. The drying time reduces by 41% to 2.99 h. Since only one shelf is loaded, the cycle is never limited by the equipment capability on account of low overall sublimation rates. The limiting factor, therefore, is the product temperature and the optimization is carried out by maintaining *T*_bot_ at the limiting product temperature of − 5°C, *T*_sh_ at 30°C, and determining the chamber pressure to obtain the highest sublimation rate possible under the given conditions.

Figure 15c shows that optimizing the shelf temperature at a constant chamber pressure of 150 mTorr leads to a further improvement in drying time which drops to 2.11 h. The cycle parameters are limited by the maximum shelf temperature of 120°C till 40% of the product is dried. This is because at 150 mTorr chamber pressure and 120°C shelf temperature, the highest sublimation flux possible does not produce a maximum product temperature which is greater than the limiting value. *T*_pr,   max_ increases continuously till it reaches the limiting value, and beyond this point, the cycle parameters become product temperature limited. The shelf temperature decreases continuously to ensure the − 5°C limit for the product is not exceeded.

Figure 15dshows the most optimal primary drying process possible for the given conditions and constraints. Both the chamber pressure and shelf temperature are variable. The figure shows that for most of the cycle, the maximum shelf temperature and maximum product temperature are both limiting factors. In other words, the most optimal chamber pressure is the one that produces the maximum sublimation flux when the shelf temperature and product bottom temperature are fixed at their maximum limiting values. The driving force for sublimation is the pressure difference between the sublimation front and the chamber. As the drying progresses, the product resistance increases, and a greater pressure difference is required to sustain the maximum sublimation flux. Consequently, the *P*_ch, opt_ reduces continuously till the minimum chamber pressure limit is reached when 83% of the product is dried. For the rest of the cycle, this minimum *P*_ch_ is the limiting factor. The total drying time is 1.96 h and the optimization leads to 62% reduction in the primary drying time when compared with the typical cycle.

**Fig. 16:**
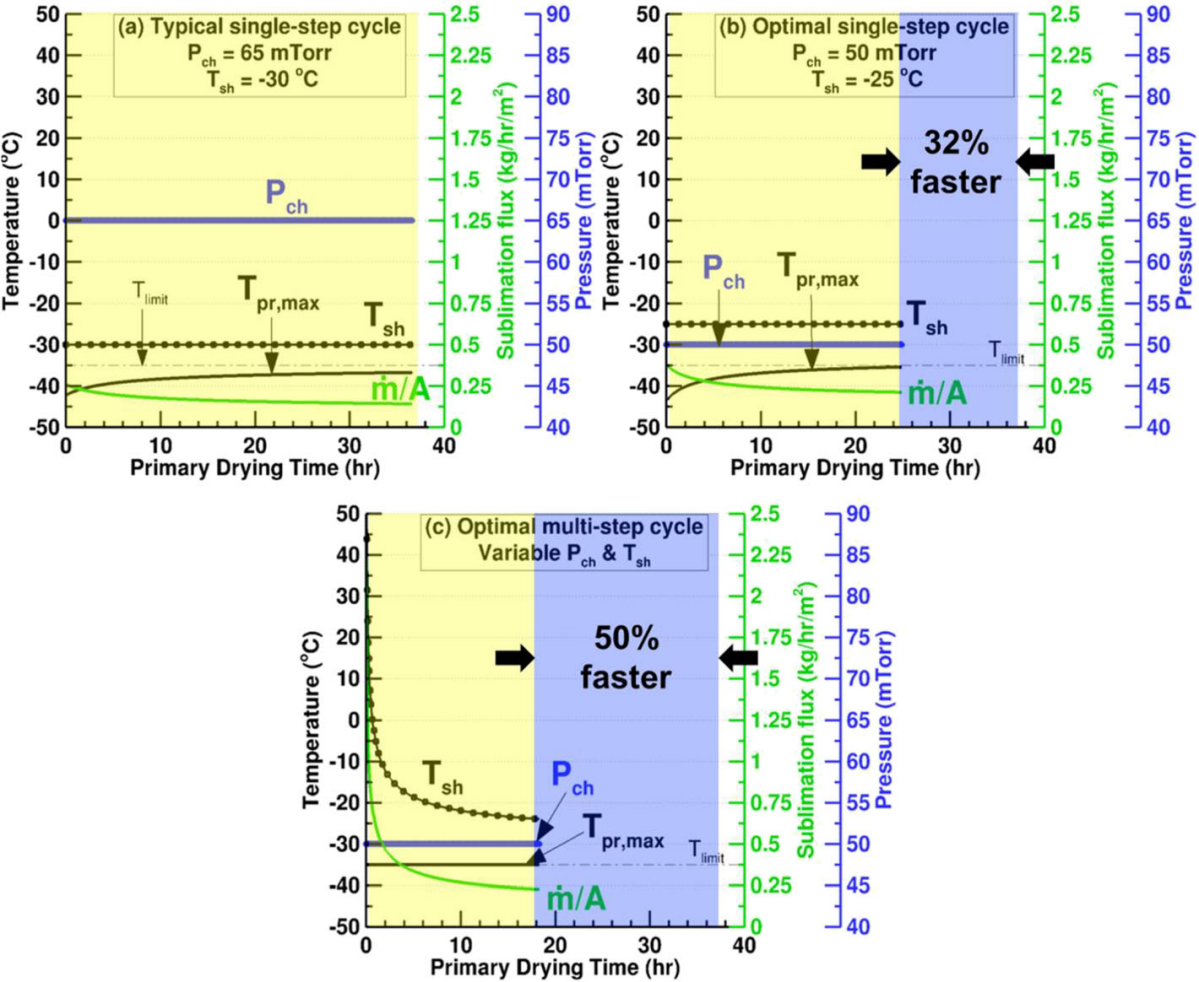
Effect of the *P*_ch_ and *T*_sh_ optimization on the primary drying parameters for 1 mL of 5% sucrose in 6R vials on one loaded shelf of REVO

Figure 16 compares two different single setpoint cycles with an optimized cycle with variable chamber pressure and shelf temperature for 2 mL of 5% sucrose in Schott 6R vials. The maximum product temperature limit in this case is set to − 35°C so as to be 3°C below the glass transition temperature for 5% sucrose (35). The product resistance for freeze drying 5% sucrose is obtained from the Cake Resistance Library in the Excel-based Lyocycle Design and Transfer Template (22) developed by Dr. Serguei Tchessalov at Pfizer Inc. The value is determined based on fitted data from Dr. Pikal’s laboratory and is listed in Table I. For typical single setpoints of 65 mTorr and − 30°C, the primary drying of one loaded shelf on Millrock REVO requires 36.64 h. Optimizing the single setpoints such that the chamber pressure and shelf temperature are maintained at the most optimal point on the cycle design space reduces the primary drying time to 24.88 h. The chamber pressure is maintained at its minimum possible value of 50 mTorr and the shelf temperature is − 25°C so that the maximum product temperature does not exceed − 35°C. Allowing for variable *P*_ch_ and *T*_*sh*_ reduces the primary drying time by half. Low chamber pressures are more suitable for sucrose cycles and the optimal value stays at its minimum value of 50 mTorr. Since the limiting product temperature is much lower than that for 5% mannitol, the shelf temperature never reaches its maximum value of 120°C. The sublimation rate is limited by the low shelf temperature and is not high enough to exceed the equipment capability. The optimal shelf temperature is the one that produces the highest sublimation flux for a product bottom temperature of − 35°C and chamber pressure of 50 mTorr.

**Fig. 17:**
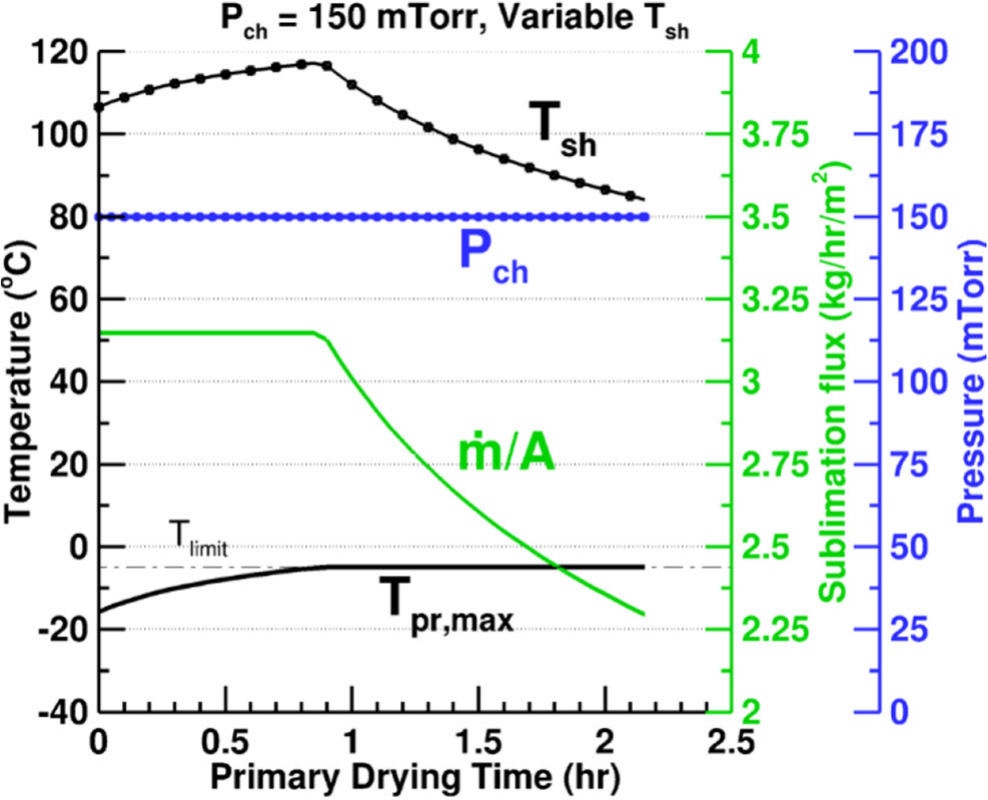
*T*_sh_ optimization at *P*_ch_ = 150 mTorr for the primary drying of 2 mL of 5% mannitol in 6R vials on four loaded shelves of REVO

In order to demonstrate the behavior of the optimizer when the equipment capability is the limiting factor, we perform a simulation for product and fill conditions similar to that in Fig. 15. However, all four shelves of the lyophilizer are loaded in this case. The chamber pressure is fixed at 150 mTorr and the shelf temperature is maintained at the most optimal value under the same constraints as before. Figure 17 shows that the sublimation flux stays constant at its limiting value till 43% of the product is dried. The shelf temperature increases till this point because the product resistance increases with time and more thermal energy is required to maintain the same sublimation flux. The maximum product temperature also increases due to the increasing shelf temperature, reaches its maximum possible value at 0.85 h, and becomes the limiting factor beyond this point. These conditions result in a 58% faster cycle than a typical one shown in Fig. 15a indicating that the optimization is equally effective under partial and full load conditions.

In all these simulations, we do not consider the effects of heat transfer non-uniformity encountered in the lyophilization chamber. We assume that the vial heat transfer coefficient and drying time are the same for all the vials loaded in the chamber and equal to those of the center vials on account of their longer drying time. A simple way for incorporating this in the simulations is to divide the total number of vials into center and edge vials and use different vial heat transfer coefficients for each set. With more accurate process monitoring using sensor networks, the measurements could be coupled with the code to determine the most optimal conditions that not only minimize the total drying time within the constraints, but also minimize the non-uniformity in the chamber. The maximum shelf temperature that can be used without product lift-off or vial blow-out must be characterized experimentally and this must be imposed as the limiting value. Analysis of the residual moisture content and specific surface area tests to evaluate cake shrinkage in addition to the experimental demonstration of variable process parameter optimization are to be included in future work.

## CONCLUSIONS

The Lyophilization Process Optimization Tool or LyoPRONTO is an open-source application that can be used to model various aspects of the lyophilization process for a given cycle and to design more efficient cycles. The existing freezing models require 2D or 3D finite element analysis of the product in the vial which is time consuming and computationally expensive. LyoPRONTO presents a novel method of applying a 0D lumped capacitance heat transfer model to predict the crystallization time and product temperature profile during the freezing process. The overprediction of the ice crystallization time when compared with experiments can be attributed to the stochastic nature of the nucleation process and the assumption of a 0D model. The results are qualitatively accurate and can quantitatively be used to design freezing cycles provided a factor of safety is included to account for the stochasticity. The 1D quasi-steady heat and mass transfer analysis of primary drying is based on existing models but using time steps instead of steps of product length. The modeling results deviate on average by 3% from experimental measurements. The tool is also capable of determining the vial heat transfer characteristics and product resistance parameters based on the experimental drying time and product temperature profile respectively which reduces the number of overall experiments required for the complete characterization of a lyophilization cycle.

LyoPRONTO can generate the primary drying design space for a given product, vial, load, and lyophilizer combination, with the most optimal setpoint of operation being the point of intersection of the equipment capability curve and the maximum allowable product temperature isotherm. Since this optimal point varies at each instant of time during a cycle, the most efficient cycle is one with variable time-dependent chamber pressure and shelf temperature profiles set at their optimal values at all times. The optimizer tool in LyoPRONTO is designed to determine such a variable profile for chamber pressure and/or shelf temperature within the constraints of the equipment, product, and practical limitations. The time interval for the variation can also be changed as an input parameter. Optimal cycles with 3 min variations in chamber pressure and shelf temperature result in a 62% reduction in the primary drying time for a 5% mannitol cycle in a laboratory scale when compared with a typical single setpoint cycle. This reduction is 50% for 5% sucrose solution and the optimization results in a significant primary drying time reduction under both partial and full load conditions.
